# The effect of procedural time on dysplasia detection rate during endoscopic surveillance of Barrett’s esophagus

**DOI:** 10.1055/a-2015-8883

**Published:** 2023-03-09

**Authors:** Mathew Vithayathil, Ines Modolell, Jacobo Ortiz-Fernandez-Sordo, Apostolos Pappas, Wladyslaw Januszewicz, Maria O’Donovan, Michele Bianchi, Jonathan R. White, Philip Kaye, Krish Ragunath, Massimiliano di Pietro

**Affiliations:** 1 Early Cancer Institute, Department of Oncology, University of Cambridge, Cambridge, United Kingdom; 2 Department of Surgery and Cancer, Imperial College London, Hammersmith Hospital, London, United Kingdom; 3 Department of Gastroenterology, Cambridge University Hospital NHS Foundation Trust, Cambridge, United Kingdom; 4 Nottingham Digestive Diseases Centre and NIHR Nottingham Biomedical Research Centre, Nottingham University Hospitals NHS Trust and University of Nottingham, Nottingham, United Kingdom; 5 Department of Gastroenterology, Hepatology and Clinical Oncology, Medical Centre for Postgraduate Education, Warsaw, Poland; 6 Department of Histopathology, Cambridge University Hospital NHS Foundation Trust, Cambridge, United Kingdom; 7 Department of Histopathology, Nottingham University Hospital NHS Foundation Trust, Nottingham, United Kingdom

## Abstract

**Background** Endoscopic surveillance of Barrett’s esophagus (BE) with Seattle protocol biopsies is time-consuming and inadequately performed in routine practice. There is no recommended procedural time for BE surveillance. We investigated the duration of surveillance procedures with adequate tissue sampling and effect on dysplasia detection rate (DDR).

**Methods** We performed post hoc analysis from the standard arm of a crossover randomized controlled trial recruiting patients with BE (≥C2 and/or ≥M3) and no clearly visible dysplastic lesions. After inspection with white-light imaging, targeted biopsies of subtle lesions and Seattle protocol biopsies were performed. Procedure duration and biopsy number were stratified by BE length. The effect of endoscopy-related variables on DDR was assessed by multivariable logistic regression.

**Results** Of 142 patients recruited, 15 (10.6 %) had high grade dysplasia/intramucosal cancer and 15 (10.6 %) had low grade dysplasia. The median procedural time was 16.5 minutes (interquartile range 14.0–19.0). Endoscopy duration increased by 0.9 minutes for each additional 1 cm of BE length. Seattle protocol biopsies had higher sensitivity for dysplasia than targeted biopsies (86.7 % vs. 60.0 %; *P* = 0.045). Longer procedural time was associated with increased likelihood of dysplasia detection on quadrantic biopsies (odds ratio [OR] 1.10, 95 %CI 1.00–1.20, *P* = 0.04), and for patients with BE > 6 cm also on targeted biopsies (OR 1.21, 95 %CI 1.04–1.40; *P* = 0.01).

**Conclusions** In BE patients with no clearly visible dysplastic lesions, longer procedural time was associated with increased likelihood of dysplasia detection. Adequate time slots are required to perform good-quality surveillance and maximize dysplasia detection.

## Graphical abstract

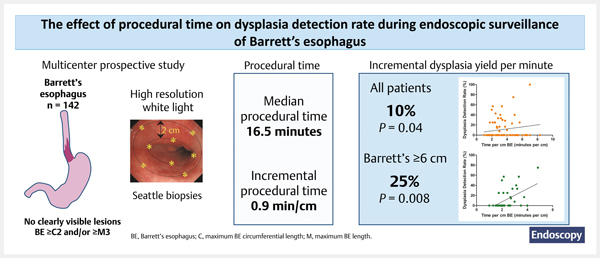

## Introduction

Patients with Barrett’s esophagus (BE) are at increased risk of esophageal adenocarcinoma (EAC), a highly lethal cancer with a 5-year overall survival lower than 20 % 1 . Endoscopic surveillance of BE is recommended by all major gastroenterology societies as a method of reducing mortality and morbidity related to EAC 2 3 4 . This recommendation is supported by several retrospective studies, which showed that patients with BE who receive regular endoscopic monitoring have reduced cancer-related mortality and lower incidence of advanced-stage cancer 5 6 7 . However, the protective effect offered by BE surveillance has not been confirmed by all studies. For example, Corley et al. showed in a case–control design that patients with BE who died from EAC had similar rates of endoscopic surveillance to patients who did not die of their BE disease 8 . The explanation for this discrepancy is likely to reside in the quality of the endoscopic surveillance, which varies across institutions and operators. For example, Verbeek et al. demonstrated that surveillance exerts a protective effect against mortality by EAC only if it is performed adequately and in line with guideline recommendations 7 .

The long procedural time required by the recommended endoscopic biopsy protocol (Seattle protocol 9 ) is among the factors explaining the low level of adherence to guideline recommendations and poor quality of endoscopic surveillance. Random biopsies are required because the pre-invasive neoplastic stages, also known as low grade dysplasia (LGD) and high grade dysplasia (HGD), can be completely invisible on both high resolution white-light endoscopy (HRWLE) and image-enhanced endoscopy such as narrow-band imaging and acetic acid chromoendoscopy 10 11 . Despite some evidence that longer inspection time is associated with higher detection of pre-invasive disease in BE 12 , guideline recommendations do not stipulate the optimal procedural time for BE surveillance. Therefore, the time allocated for upper gastrointestinal (GI) endoscopy is often insufficient for adequate BE surveillance. As a result, adherence to Seattle protocol biopsies is suboptimal, particularly for very long segments of BE, which also carry a higher risk of neoplastic progression 13 14 .

In order to gain insight on the optimal duration of standard endoscopic surveillance with strict Seattle protocol biopsies, we performed a post hoc analysis of data from a multicenter randomized crossover endoscopy trial, in which patients with BE and no evidence of macroscopically visible neoplastic lesions were randomized to receive either standard HRWLE with Seattle protocol biopsies or image-enhanced endoscopy with autofluorescence imaging and probe-based confocal laser endomicroscopy and targeted biopsies.

The primary objective of the study was to assess the adequate procedural time of BE surveillance with strict Seattle protocol biopsies. The secondary objective was to correlate detection of BE-related neoplasia with number of biopsies (random and/or targeted), procedural time, and endoscopist experience.

## Methods

### Study design

Patients undergoing Barrett’s surveillance from the ACE-B trial 15 were included in the current post hoc study. The ACE-B trial was a prospective, randomized, crossover study across two tertiary referral centers where patients underwent standard endoscopy (HRWLE plus Seattle protocol biopsies) and experimental endoscopy (autofluorescence imaging plus probe-based confocal laser endomicroscopy plus targeted biopsies) in two separate procedures performed 6–12 weeks apart. The study was approved by the Cambridgeshire Research Ethics Committee (09/H0308/118). For the current post hoc analysis, we analyzed whole cohort data from endoscopies performed in the standard arm of the trial only. During the ACE-B trial, whilst performing the standard arm endoscopy, endoscopists were blinded to the results of the experimental arm of the study and to pre-trial endoscopy and histology results.

### Participants

Inclusion criteria were patients aged over 18 years with BE maximum circumferential length of ≥ 2 cm and/or maximum BE length of ≥ 3 cm (C2 and/or M3) diagnosed on pre-trial endoscopy (as per Prague Classification 16 ) who were referred for surveillance of nondysplastic BE or assessment of flat dysplasia. Exclusion criteria were: evidence of macroscopic lesions on HRWLE clearly in keeping with BE-related neoplasia; previous evidence of BE-related neoplasia visible on endoscopy; previous histological evidence of EAC; esophagitis (Los Angeles grade ≥ B); previous esophagectomy; known allergy to fluorescein; severe or uncontrolled asthma; coagulopathy or anticoagulant/antiplatelet therapy for high risk conditions; active or severe cardiopulmonary disease; decompensated liver disease.

### Endoscopic procedure

Patients received two endoscopy procedures during the trial period. In the standard arm, patients underwent HRWLE with diagnostic biopsies according to the gold standard (Seattle protocol). Briefly, the endoscopists were asked to perform a complete upper GI endoscopy with intubation to the second part of the duodenum, full photo documentation, and clinically indicated biopsy of non-esophageal lesions. Procedures were performed with FQ260Z, HQ290, or H290Z endoscopes (Olympus, Tokyo, Japan). For BE inspection, the BE mucosa was first inspected throughout the length of the BE segment. The presence of subtle lesions was not an exclusion criterion for the study; subtle lesions were defined as flat (Paris 0-IIb) lesions on non-magnified HRWLE, which were not judged by the endoscopist as obviously in keeping with BE neoplasia. These lesions received two targeted biopsies to minimize sampling error directed by HRWLE only. After inspection and targeted biopsies, random biopsies were taken at every 2 cm along the BE length. Endoscopists performing procedures within the standard arm were not allowed to use image-enhanced techniques, such as narrow-band imaging or acetic-acid chromoendoscopy. In the experimental arm, esophageal mucosa was inspected using HRWLE, followed by autofluorescence imaging and probe-based confocal laser endomicroscopy. At least two biopsies were taken from areas that were positive on autofluorescence imaging or had subtle changes on HRWLE. The full experimental protocol and data have been described previously 15 . All endoscopists had a clinical or research interest in BE and had either completed their specialist training (consultant group) or were trainees who could independently perform upper GI endoscopy and had received extensive training in BE-related neoplasia detection (independent trainee group).

### Biopsy and histology

Tissue biopsies were fixed in formalin and embedded in paraffin for histopathological assessment. Targeted and random biopsies were reviewed by a GI pathologist with extensive expertise in BE in accordance with the Vienna classification 17 . Findings of dysplasia, including indefinite for dysplasia, were reviewed by a second expert GI pathologist from the other institution, with consensus diagnosis achieved for discordant cases. Indefinite for dysplasia was considered negative for definite dysplasia. In the standard arm of the trial, p53 immunohistochemistry was performed at the discretion of pathologists in order to support a diagnosis of dysplasia, as per the standard of care. The gold standard diagnosis included histopathologic diagnoses from targeted and random biopsies taken within the standard arm. In a separate analysis we also included the histopathologic diagnosis from targeted biopsies taken in the experimental arm (see results section).

### Procedure time

The time taken to perform surveillance endoscopy was defined as the time interval between insertion of the endoscope to the time of patient extubation.

### Study outcomes

Maximum BE lengths were classified into different strata: 2–5 cm, 6–10 cm, ≥ 11 cm. Time to perform endoscopy for each BE length strata was determined. To assess adherence to the Seattle protocol, the number of quadrantic biopsies taken per 2 cm length of BE was calculated by dividing total number of biopsies by half the maximum length of BE; in this analysis, four biopsies/2 cm is the standard. To assess whether operator experience influenced dysplasia detection rate (DDR) and endoscopy duration, endoscopists were divided into consultant and independent trainee groups, based on their training grade. DDR and endoscopy duration were determined for the consultant and independent trainee groups, respectively. Finally, the impact of procedural time on DDR overall, and DDR by random and targeted biopsies separately, was assessed as a function of each additional minute of procedural time.

### Statistical analysis

Median, interquartile range (IQR), and range were calculated for endoscopy duration at different BE length strata. Mean and range were determined for number of quadrantic biopsies. Comparison of endoscopy duration and number of biopsies at different BE length strata were compared using a one-way analysis of variance. *P* values of less than 0.05 were considered significant.

The effect of BE length on endoscopy duration was determined using linear regression. Endoscopy duration and number of Seattle protocol biopsies per 2 cm of BE length were compared between consultant and independent trainee groups using unpaired Student’s *t* test. Detection of dysplasia was compared between these two groups using chi-squared analysis.

We aimed to determine the effect of endoscopy duration on dysplasia detection. Both endoscopist experience 18 19 and BE length 20 have been shown to influence dysplasia detection. These factors may also impact endoscopy duration. Therefore, we performed multivariable logistic regression to determine the effect of endoscopy duration on DDR, adjusting for endoscopist experience (consultant vs. independent trainee) and BE length as covariables. Adjusted odds ratios (OR) for DDR were determined, with 95 %CIs. ORs presented were estimates for relative risk. Analysis was conducted for overall, targeted, and Seattle protocol biopsies. This effect was examined within the overall cohort and by dividing the cohort based on the BE length (BE shorter than or equal to the mean and longer than the mean). Linear regression was used to determine the effect of procedural time per maximum length of BE on DDR. Beta coefficients from linear regression models were used to determine the effect of additional procedural time per BE length on DDR. Beta coefficients were defined as the degree of change in DDR for every 1-minute change of procedural time.

All statistical analyses were conducted using SAS 9.4 software (Cary, North Carolina, USA).

## Results

**Fig. 1:**
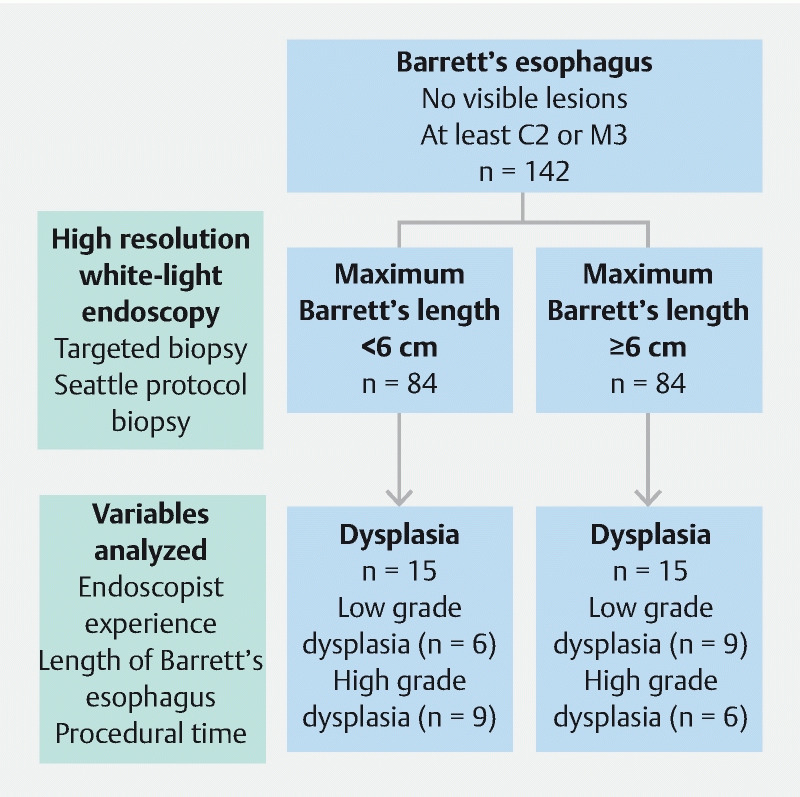
Study flow chart and breakdown of low grade and high grade dysplasia in patients with Barrett’s esophagus maximum length < 6 cm and ≥ 6 cm.

A total of 142 eligible patients completed the standard arm of the trial (HRWLE plus Seattle protocol biopsies) between May 2017 and October 2019. Baseline characteristics are shown in Table 1 . Overall, 15 patients (10.6 %) had HGD/intramucosal carcinoma (IMC) and 15 (10.6 %) had LGD ( Fig. 1 ). A mean of 2.5 targeted biopsies and 11.3 nontargeted quadrantic biopsies were taken per patient. Targeted biopsies detected dysplasia in 18 patients (7 LGD and 11 HGD/IMC) and quadrantic biopsies detected dysplasia in 26 patients (15 LGD and 11 HGD/IMC); 14 patients (46.7 %) with dysplasia had positive biopsies on both targeted and quadrantic biopsies (7 LGD and 7 HGD/IMC). A total of 12 patients (40.0 %) had dysplasia detected solely on Seattle protocol biopsies, whereas 4 patients (13.3 %) received a diagnosis of dysplasia exclusively on targeted biopsies. Overall, Seattle protocol biopsies had a higher sensitivity for detecting any grade of dysplasia compared with targeted biopsies (86.7 % vs. 60.0; *P* = 0.045). Detection of HGD/IMC was comparable between random and targeted biopsies (73.3 % vs. 73.4; *P* > 0.99). When considering the highest grade of dysplasia detected in each patient from both arms of the ACE-B trial (trial histology), the Seattle protocol again showed a higher sensitivity for detection of any grade of dysplasia compared with targeted biopsies (71.4 vs. 48.6 %; *P* = 0.03).

**Table 1:** Patient demographics.

Total number of patients, n	142
Demographic details	
Age, mean (range), years	67.3 (38.0–89.0)
Male sex, n (%)	104 (73.2)
Length of Barrett’s segment	
Maximum length, mean (range),	5.6 (2–15)
Circumferential length of Barrett’s, mean (range)	3.5 (0–13)
Overall histological diagnosis, n (%)	
NDBE	106 (74.6)
Indefinite for dysplasia	6 (4.2)
LGD	15 (10.6)
HGD/IMC	15 (10.6)
Tissue biopsies	
Total number of tissue biopsies, n	1764
Targeted biopsies, n (%)	175 (9.9)
Seattle protocol biopsies, n (%)	1589 (90.1)
Number of tissue biopsies per patient, mean (range)	12.4 (2–33)
Targeted biopsies, mean (range)	2.5 (1–9)
Seattle protocol biopsies, mean (range)	11.3 (2–31)

NDBE, nondysplastic Barrett’s esophagus; LGD, low grade dysplasia; HGD, high grade dysplasia; IMC, intramucosal carcinoma.

### Procedural time

**Fig. 2:**
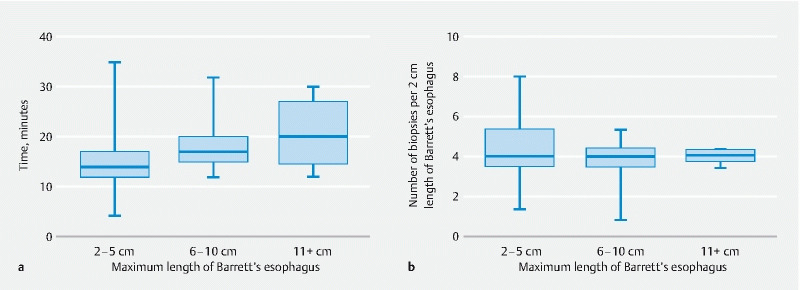
Endoscopy duration and number of biopsies according to Barrett’s esophagus (BE) length. Median, interquartile range, maximum and minimum durations are shown for each length strata. **a** Surveillance endoscopy duration. Endoscopy duration increased with increasing BE length (one-way analysis of variance [ANOVA]). **b** Number of Seattle protocol biopsies per 2 cm of BE. Number of biopsies decreased with increasing BE length (one-way ANOVA).

The median time to perform HRWLE with Seattle protocol biopsies was 16.5 minutes (IQR 14.0–19.0 minutes). The time to perform BE surveillance endoscopy for increasing lengths of BE is shown in Fig. 2a . As expected, increasing BE length was associated with a significant increase in endoscopy duration ( *P* < 0.001). The median procedural time in the BE length strata 2–5 cm, 6–10 cm, and ≥ 11 cm was 14.0 minutes (IQR 12.0–17.0), 17.0 minutes (IQR 15.0–20.0), and 20.0 minutes (IQR 14.5–27.0), respectively. In linear regression, there was a significant association between BE length and endoscopy duration, with each additional 1 cm of BE increasing the duration of endoscopy by 0.9 minutes ( *P* < 0.001).

### Adherence to the Seattle protocol

To evaluate adherence to the Seattle protocol, the number of quadrantic biopsies taken per 2 cm of BE was assessed. The mean number of biopsies in the different strata 2–5 cm, 6–10 cm, and ≥ 11 cm was 4.3 (range 1.3–8.0), 3.8 (range 0.8–5.3), and 4.0 (range 3.4–4.3), respectively. Even though adherence to the Seattle protocol was high across different strata and did not drop below the recommended standard, we found variation within the strata and a small but statistically significant decrease in the mean number of biopsies per 2 cm of BE with increasing length ( *P* = 0.047) ( Fig. 2b ).

### Endoscopist experience

We evaluated whether endoscopist training grade influenced endoscopy performance. There was no difference in sensitivity for dysplasia detection from targeted (consultant 45.5 % vs. independent trainee 68.4 %; *P* = 0.22) and quadrantic (consultant 100 % vs. independent trainee 79.0 %; *P* = 0.10) biopsies. Similarly, we observed no difference between consultant and independent trainee endoscopists in median endoscopy duration (15.0 [IQR 13.0–19.0] vs. 16.0 [IQR 14.0–18.5] minutes; *P* = 0.21) or adherence to the Seattle protocol (4.2 vs. 4.1 biopsies per 2 cm BE; *P* = 0.43).

### Dysplasia detection rate

**Fig. 3:**
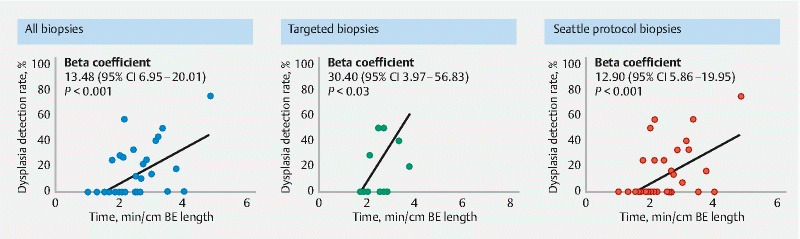
Linear regression for dysplasia detection rate (DDR) and endoscopy duration per maximum Barrett’s esophagus (BE) length for all biopsies, targeted biopsies, and Seattle protocol biopsies in patients with BE ≥ 6 cm. Linear regression beta coefficients are shown with 95 %CIs and *P* value.

We then looked at the impact of endoscopy duration on DDR. Both DDR and endoscopy duration may be affected by BE length and endoscopist experience. Therefore, we performed multivariable logistic regression with endoscopy duration adjusting for BE length and endoscopist experience. As show in Table 2 , duration of endoscopy significantly increased the likelihood of dysplasia detection from quadrantic biopsies (OR 1.10, 95 %CI 1.00–1.20; *P* = 0.04). The magnitude of this effect from targeted biopsies was smaller (OR 1.05, 95 %CI 0.95–1.15). For BE length ≥ 6 cm (based on the mean BE length in the cohort), duration of endoscopy significantly increased the likelihood of dysplasia detection from targeted biopsies (OR 1.21, 95 %CI 1.04–1.40; *P* = 0.01), Seattle protocol biopsies (OR 1.25, 95 %CI 1.06–1.47; *P* = 0.008), and overall (OR 1.22, 95 %CI 1.05–1.43; *P* = 0.01) ( **Table 1 s** in the online-only Supplementary material). This association was not observed for patients with BE lengths of < 6 cm ( **Table 2 s** ). For HGD/IMC, increasing endoscopy duration was not associated with increased DDR for patients with any BE length ( **Table 3 s** ). However, for BE lengths ≥ 6 cm, longer procedure duration was associated with increased HGD/IMC detection for all biopsies (OR 1.22, 95 %CI 1.02–1.46; *P* = 0.03) and Seattle protocol biopsies (OR 1.29, 95 %CI 1.03–1.60; *P* = 0.03) ( **Table 4 s** ). In linear regression analysis, there was a significant increase in DDR for all biopsies (beta coefficient 13.48, 95 %CI 6.95–20.01; *P* < 0.001), targeted biopsies (beta coefficient 30.40, 95 %CI 3.97–56.83; *P* = 0.03), and Seattle protocol biopsies (beta coefficient 12.90, 95 %CI 5.86–19.95; *P* < 0.001) for each additional 1 minute of procedural time spent for patients with BE length ≥ 6 cm ( Fig. 3 ). No significant association was seen on the entire cohort across all BE lengths ( **Fig. 1 s** ).

**Table 2:** Multivariable logistic regression for detection of dysplasia obtained from targeted and Seattle protocol biopsies.

	Dysplasia detected		
	From all biopsies	From targeted biopsies	From Seattle protocol biopsies
Incremental yield of dysplasia per additional minute, OR 1 (95 %CI)	1.06 (0.97–1.15) *P* = 0.20	1.05 (0.95–1.15) *P* = 0.37	1.10 (1.00–1.20) *P* = 0.04

OR, odds ratio.

1 Adjusted OR for endoscopy duration, adjusted for endoscopist experience and Barrett’s esophagus maximum length from multivariable logistic regression.

## Discussion

This study demonstrated that the median duration for an adequate BE endoscopic surveillance procedure was 16.5 minutes, no difference between consultant endoscopists and independent trainees who had completed specialist training. In addition, we found evidence that the duration of the endoscopy procedure correlated with DDR.

Despite unanimous agreement among specialist guidelines in recommending endoscopic surveillance for BE, there is still some debate about the absolute impact of this intervention on patient outcomes. Although most retrospective studies found that surveillance correlated with diagnosis of cancer at an earlier stage and a reduction in mortality from EAC, the evidence suggests that the effect is smaller than expected 6 7 . For example, El-Serag et al. found that although BE patients undergoing surveillance had a 50 % reduction in the hazard ratio for EAC-related death, the rates of 3-year and 5-year mortality from EAC in the surveillance group were 33.2 % and 48.8 %, respectively, suggesting that in this study many patients diagnosed with EAC on surveillance still eventually die of BE-related cancer 6 . Similarly, in a Dutch retrospective study, even though the hazard ratio for EAC mortality in patients receiving adequate surveillance was 0.76 (95 %CI 0.64–0.92) compared with those who were not monitored for BE, the 2-year mortality rate in patients receiving adequate surveillance was significant at 38 % 7 . Of note, adequacy of surveillance was assessed based on the time interval between cancer diagnosis and previous endoscopy and not based on adherence to the Seattle protocol. Overall, these data suggest that, although adequate endoscopic procedures are essential to maximize the benefit from surveillance, there is an opportunity to further reduce EAC-related mortality in standard clinical practice and to diminish the rate of post-endoscopy EAC Barrett’s neoplasia 21 22 , which is estimated to represent between 2 and 14 % of Barrett’s-related cancers.

BE-related neoplasia can be subtle and focal; therefore, a high quality endoscopic procedure is essential for optimization of dysplasia diagnosis and initiation of timely therapeutic interventions. Although intense research is being undertaken to investigate the impact of image-enhanced endoscopy on DDR, there is no definite evidence that image-enhanced endoscopy improves DDR in BE; therefore, current European and British guidelines recommend against routine use of image-enhanced endoscopy in routine practice 2 3 . Hence, the current gold standard for surveillance is HRWLE with Seattle protocol biopsies. However, this protocol is challenging to perform in routine practice, as BE surveillance is generally performed during standard endoscopy lists, with time allocation per procedure as short as 15–20 minutes, which has to include cannulation, full endoscopic examination with biopsies, post-procedural checks, and reporting time. Our study clearly demonstrated that the average duration of an adequate BE surveillance procedure with full endoscopic inspection and Seattle protocol biopsies was over 16 minutes, which is not compatible with the standard time allocated for upper GI procedures and is significantly longer than the minimum standard of 7 minutes recommended for diagnostic upper GI endoscopy by the European Society of Gastrointestinal Endoscopy Quality Improvement Initiative 23 . The British Society of Gastroenterology recommends that a standard diagnostic endoscopy is allocated a minimum time slot of 20 minutes, increasing as appropriate for surveillance or high risk conditions 24 . Our data suggest that for BE lengths up to 5 cm, a 30-minute slot should be allocated, whereas for BE longer than 5 cm, a “double slot” should be preferred (equivalent to 40 minutes), regardless of endoscopist level of experience. Although nurse endoscopists were not included in our study, it is likely that our findings would extend to this category too.

Previous data suggest that adherence to the Seattle protocol drops for long segments of BE 13 14 . Even though our current data derive from a rigorous prospective protocol, we observed a slight but significant drop in the average number of biopsies adjusted for length of BE. This drop is expected to become numerically more significant in routine practice outside of a well-controlled research protocol. The implication of this reduction in number of biopsies can be seen in the effect of duration of endoscopy on DDR. Interestingly, while we did not see an impact of procedural time on DDR by targeted biopsies, we found that a longer duration of endoscopy increased detection of dysplasia from random biopsies, suggesting that sufficient time allocation allows maximization of tissue sampling with positive impact on DDR. The greater magnitude of the correlation between procedural time and DDR from targeted biopsies in patients with BE length of ≥ 6 cm can be explained by challenges related to endoscopic assessment of very long segments of BE and the higher likelihood that endoscopists will reduce the time of inspection per centimeter of BE in these cases. We did not find that increasing endoscopy duration was associated with a higher likelihood of HGD/IMC detection. This may be due to level of endoscopist experience in the trial and to the exclusion of patients with clearly visible lesions on HRWLE, which has been shown to have a positive predictive value of up to 47 % for HGD/IMC 25 .

Overall, our findings are in agreement with previous evidence from another randomized trial showing a correlation between BE inspection time and DDR 12 . In this study, endoscopists with an average BE inspection time of 1 minute or more were more likely to detect suspicious lesions, although the effect of inspection time > 1 minute on DDR was not statistically significant. However, the authors did not perform a separate analysis for patients with very long BE segments. In addition, as patients with visible neoplastic lesions were allowed in this study, it is possible that at least a proportion of the inspection time was due to endoscopic characterization of a visible lesion. Finally, HRWLE and image-enhanced endoscopy were performed within the same procedure, which could affect the procedural time. Overall, our study would support the concept of a procedural time of an additional 1 minute per centimeter of BE.

This study has several strengths. It was a prospective study with good correlation between endoscopic and histopathologic findings, and precise recording of the procedural time. Endoscopy duration included full inspection of the upper GI tract, which is recommended in general practice. As we excluded patients with obvious neoplastic lesions, these data are relevant to the general surveillance population, where endoscopists are not expected to encounter visible lesions in the majority of cases. However, there are potential limitations. This study was a post hoc analysis of a trial assessing the utility of autofluorescence imaging, probe-based confocal laser endomicroscopy, and biomarkers of dysplasia detection. The correlation between procedural time and DDR was not a primary outcome of the original clinical trial, and therefore the current study may not be adequately powered for the outcomes analyzed. Further specific prospective studies on an unselected BE cohort may be required to fully determine the optimal procedural time for dysplasia detection. No image-enhanced endoscopy was allowed in the standard arm of the study. All current endoscopy technologies feature button switch electronic chromoendoscopy, which is easy to perform and increasingly used in standard practice. While use of image-enhanced endoscopy could have had a positive impact on DDR, it is not recommended by the European and British guidelines in routine practice, and therefore was not included in the standard arm of the trial. All endoscopists in this study were BE expert endoscopists from two centers only, including trainees who received extensive training in BE-related neoplasia detection. Therefore, the results of this study cannot be directly translated to practices with endoscopists who are less experienced in BE surveillance. However, as the positive effect of procedure duration on DDR was seen predominantly on Seattle protocol biopsies, this effect is likely to be applicable to standard practice and a wider range of operators. Finally, we did not record the esophageal inspection time separately, only the overall duration of the procedure. Therefore, our data do not allow us to derive definite information on the BE inspection time. However, we were able to show that in an adequate procedure, 0.9 minutes were necessary per additional centimeter of BE length and that DDR increased for each 1 minute of procedural time, providing some indication about the average procedural time required based on the extent of the disease.

In conclusion, it is important that time allocation for BE surveillance endoscopy is standardized in order to allow endoscopists to perform adequate procedures. The full beneficial impact of surveillance on patient outcome is only achievable if endoscopists are allowed to spend sufficient time inspecting and sampling the diseased mucosa. Future guidelines must consider this important issue and provide clear recommendation on the optimal duration of BE surveillance procedures.

## Tables 1 s–4 s, Fig. 1 s

Supplementary material Zusatzmaterial
