# Supplementary material for: The effect of procedural time on dysplasia detection rate during endoscopic surveillance of Barrett’s esophagus
Source: Endoscopy. 2023 Mar 9;55(6):491–8. doi: 10.1055/a-2015-8883 (PMC10212647; doi:10.1055/a-2015-8883)
Supplement: Supplementary file 1 — Supplementary material [file 22108supmat_10-1055-a-2015-8883.pdf]

Supplementary material

The effect of procedural time on dysplasia detection rate during endoscopic surveillance of Barrett's esophagus

Mathew Vithayathil, Ines Modolell, Jacobo Ortiz-Fernandez-Sordo, Apostolos Pappas, Wladyslaw Januszewicz, Maria O'Donovan, Michele Bianchi, Jonathan White, Philip Kaye, Krish Ragunath, Massimiliano di Pietro

Supplementary material

Table 1s Multivariable logistic regression for detection of dysplasia obtained from targeted and Seattle protocol biopsies for maximal BE length 6cm and greater

	Dysplasia from all biopsies	Dysplasia from targeted biopsies	Dysplasia from Seattle protocol biopsies
**Incremental yield of dysplasia per additional minute (Odds Ratio)**	1.22 (1.05, 1.43) *p*=0.01	1.21 (1.04, 1.40) *p*=0.01	1.25 (1.06, 1.47) *p*=0.008

Adjusted odds ratio (95% confidence interval) for endoscopic duration, adjusted for endoscopist experience and Barrett's Esophagus maximal length from multivariable logistic regression with *p* values shown.

Table 2s Multivariable logistic regression for detection of dysplasia obtained from targeted and Seattle protocol biopsies for maximal BE length less than 6cm

Adjusted odds ratio (95% confidence interval) for endoscopic duration, adjusted for endoscopist experience and Barrett's Esophagus maximal length from multivariable logistic regression with *p* values shown.

	Dysplasia from all biopsies	Dysplasia from targeted biopsies	Dysplasia from Seattle protocol biopsies
**Incremental yield of dysplasia per additional minute (Odds Ratio)**	0.88 (0.75, 1.04) *p*=0.13	0.86 (0.71, 1.04) *p*=0.12	0.94 (0.80, 1.11) *p*=0.46

Table 3s Multivariable logistic regression for detection of high-grade dysplasia/intramucosal carcinoma obtained from targeted and Seattle protocol biopsies

	Dysplasia from all biopsies	Dysplasia from targeted biopsies	Dysplasia from Seattle protocol biopsies
**Incremental yield of dysplasia per additional minute (Odds Ratio)**	1.01 (0.90, 1.13) *p*=0.87	0.93 (0.79, 1.09) *p*=0.38	1.06 (0.94, 1.19) *p*=0.36

Adjusted odds ratio (95% confidence interval) for endoscopic duration, adjusted for endoscopist experience and Barrett's Esophagus maximal length from multivariable logistic regression with *p* values shown.

Supplementary material

Table 4s Multivariable logistic regression for detection of high-grade dysplasia/intramucosal carcinoma obtained from targeted and Seattle protocol biopsies for maximal BE length 6cm and greater

	Dysplasia from all biopsies	Dysplasia from targeted biopsies	Dysplasia from Seattle protocol biopsies
**Incremental yield of dysplasia per additional minute (Odds Ratio)**	1.22 (1.02, 1.46) *p*=0.03	1.06 (0.85, 1.34) *p*=0.58	1.29 (1.03, 1.60) *p*=0.03

Adjusted odds ratio (95% confidence interval) for endoscopic duration, adjusted for endoscopist experience and Barrett's Esophagus maximal length from multivariable logistic regression with *p* values shown.

Supplementary material

Fig. 1s Linear regression for dysplasia detection rate (DDR) and endoscopic duration per maximal BE length for targeted, Seattle protocol and all patients

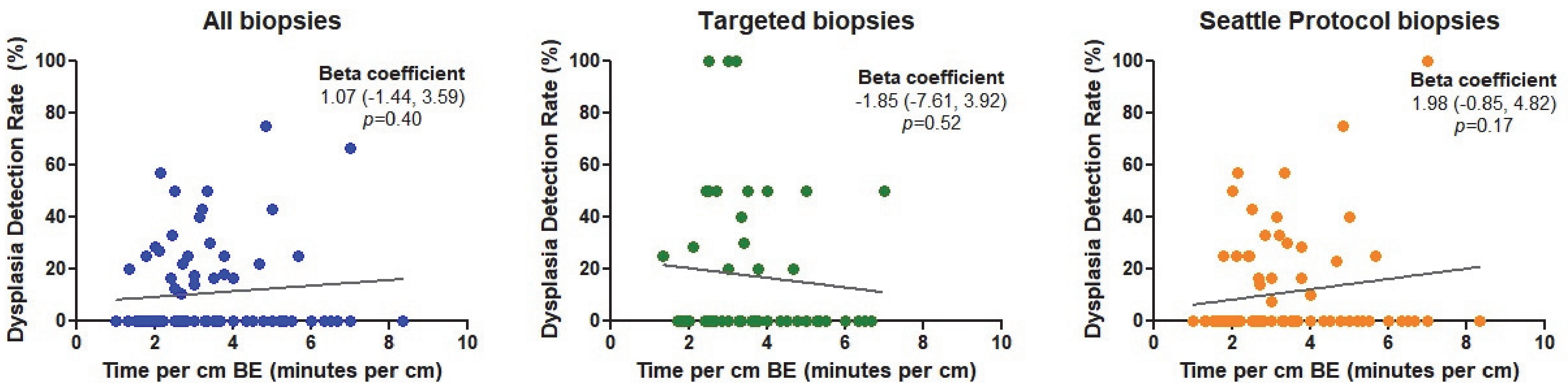

Linear regression beta coefficient shown with 95% confidence interval and *p* value.
